# Types of fathering insufficiency and their association with children's emotional well being: a latent profile

**DOI:** 10.1186/s40359-026-05460-y

**Published:** 2026-08-29

**Authors:** Shi-Min Chen, Qi-Yi Lin, Li-Li Wang, Juan Zhang, Pei-Zhen Sun

**Affiliations:** 1 Department of Psychology, Huaiyin Normal University, Huai’an, China; 2 Department of Psychology, Jiangsu Normal University, Xuzhou, China

**Keywords:** Fathering insufficiency, Paternal functioning, Emotional well-being, Latent profile analysis, Children

## Abstract

**Background:**

Father absence has become an increasingly concerning social issue. The physical absence of fathers often leads to a lack of paternal functioning, but the two are not entirely equivalent. Therefore, this study coined the term “fathering insufficiency” to describe insufficiency in paternal functioning.

**Objective:**

This study had three objectives: (1) to identify the types of fathering insufficiency using latent profile analysis; (2) to explore the proportion of fathering insufficiency in different family structures; and (3) to compare differences in children’s emotional well-being across different types of fathering insufficiency.

**Methods:**

A total of 3,199 junior middle school students were surveyed using the Inventory of Father Involvement (short version), the Inventory of Mother Involvement, and the Emotional Experience of Well-being Questionnaire.

**Results:**

Children were categorized into four classes: moderate parental functioning (M), high parental functioning (H), mild parenting insufficiency (MI), and fathering-lack-high-mothering (L-H). Both the MI and L-H classes were characterized as experiencing fathering insufficiency. The proportion of fathering insufficiency observed in the present sample was 21.4% in dual-parent co-resident families, 29.9% in left-behind families, and 66.1% in divorced families. The emotional well-being of children in the L-H class was significantly lower than that in the M class, but significantly higher than that in the MI class.

**Conclusions:**

Both the MI and L-H classes exhibited characteristics of fathering insufficiency, with different patterns showing distinct associations with children’s emotional well-being. Parenting programs can offer family education on fathering insufficiency. Fathers should use fragmented time and digital tools to raise engagement. Couples need constructive conflict management and careful divorce consideration.

**Supplementary Information:**

The online version contains supplementary material available at https://doi.org/10.1186/s40359-026-05460-y.

## Introduction

Father absence has become an increasingly prominent social concern with socioeconomic development. This phenomenon is particularly pronounced in left-behind families and single-parent households. The causes of father absence differ between these two family structures. According to role theory, fathers and mothers fulfill distinct functions within the family system: fathers typically serve as primary breadwinners, while mothers assume greater responsibility for childcare and domestic management [1–3]. In left-behind families, fathers often work away from home for extended periods to provide financial support, resulting in physical separation from their children. In single-parent households, mothers are more frequently granted primary custody following a divorce, given their typical role as the primary provider of daily child care. According to relevant surveys, this arrangement occurs in roughly three out of four cases [4, 5]. This frequent loss of paternal custody rights consequently leads to father absence in children’s lives.

The physical absence of fathers often leads to a lack of paternal functioning for their children, but the two are not entirely equivalent. Despite the presence of physical absence, the extent of the lack in paternal functioning varies among different fathers. Therefore, researchers have proposed other terms to describe the insufficiency in paternal functioning, such as “father-love absence” [6]. However, neither the term “father absence” nor “father-love absence” adequately captures the phenomenon of insufficient paternal functioning. This study proposes the term “fathering insufficiency” to more accurately describe inadequate paternal functioning, for two main reasons. First, the word “fathering” emphasizes fathers’ practical roles and caregiving behaviors [7, 8], rather than merely their paternal identity. Second, though absence, insufficiency, lack, inadequacy and deficiency all mean shortage, they differ semantically. Absence denotes total nonexistence; insufficiency and lack describe quantitative scarcity, with lack implying near-total absence. In contrast, inadequacy and deficiency stress qualitative defects or unmet standards. Since this study focuses on quantitative gaps in paternal functioning, “insufficiency” is chosen to align with its aims.

When examining fathering insufficiency, several research questions emerge. First, what do paternal functions include? It is important to note the distinction between paternal functions and paternal functioning. Paternal functioning refers to the dynamic process of carrying out paternal functions. Parental functioning consists of paternal functioning and maternal functioning. Furthermore, paternal functioning and fathering, as well as maternal functioning and mothering, share similar connotations. In the present study, these terms are regarded as equivalent constructs that can be used interchangeably. Second, insufficiency is relative to a certain reference criterion. What should serve as the criterion for fathering insufficiency, and how to assess it? Third, what is the overall proportion of fathering insufficiency, and how does its proportion differ across various family structures? Fourth, what is the association between fathering insufficiency and children’s mental health? The following sections review previous research on these questions, identify existing limitations, and outline the approach adopted in this study.

### Paternal functions

This study attempts to analyze the specific content of paternal functions from the perspective of children’s need fulfillment. First, a certain level of financial income serves as the foundation for meeting various needs of family members, making the provision of economic support the most fundamental function of a father. Second, as children have physiological needs such as eating and clothing, fathers bear the responsibility of providing daily care. Third, children encounter various difficulties and setbacks, which lead to negative emotions in the course of their growth [9, 10]. They need comfort, encouragement, and recognition, thus fathers have the role of providing emotional support. Fourth, since acquiring knowledge and skills is a crucial developmental task for children, fathers are responsible for providing academic support and facilitating their skill development [11]. Fifth, to help children understand social norms and successfully integrate into society, fathers need to implement appropriate discipline education. Sixth, as children grow older and become increasingly concerned about the future [12], they often experience uncertainty regarding their development; therefore, fathers have the function of assisting their children in future planning. In summary, fathering functions comprise at least the following six aspects: economic support, daily care, emotional support, academic support, discipline education, and future planning.

### Reference criterion and assessment of fathering insufficiency

Insufficiency is defined relative to a specific reference criterion. Father absence is assessed using the dual-parent co-resident family as the reference. But what should serve as the reference criterion for fathering insufficiency? Previous studies have not yet explored this issue. Based on the principle of normal distribution, fathers with a moderate level of paternal functioning constitute the majority of the distribution, reflecting the prevalent state of fathering practices in most households. Therefore, adopting moderate paternal functioning as the reference criterion for judging fathering insufficiency, and treating paternal functioning below this level as indicative of fathering insufficiency, hold certain rationality from both statistical and social‑practice perspectives.

This study intended to employ latent profile analysis (LPA) to identify varying levels of paternal functioning. LPA divides the population into several latent categories based on a set of indicators, maximizing homogeneity within categories and heterogeneity between categories [13]. Each category possesses distinct attributes. The classification is determined through fit statistics and significance tests. By probabilistically assigning individuals to categories, LPA accounts for classification uncertainty and measurement error, thereby offering a highly precise method of categorization.

### Prevalence of fathering insufficiency

Previous studies have primarily investigated the prevalence of physical father absence, particularly that caused by parental divorce. For example, data from the Avon Longitudinal Study of Parents and Children (ALSPAC) revealed that the prevalence of father absence due to parental divorce among children in the UK was 24.3%, with 17.7% of children experiencing father absence between the ages of 0 and 5, and 6.6% between the ages of 6 and 10 [14]. The National Longitudinal Study of Adolescent to Adult Health reported that the prevalence of father absence due to parental divorce among Americans aged 24–34 was as high as 38%, with 14% of participants experiencing parental divorce before birth, 9% between ages 0 and 5, 12% between ages 6 and 13, and 3% between ages 14 and 18 [15].

Compared with the findings above, the prevalence of father absence due to parental divorce among Chinese children is relatively lower, at 19.8% [16]. However, it is worth noting that the prevalence of paternal functional insufficiency is higher than that of physical father absence. Wang (2021) conducted a survey of 21,716 parents, identifying fathering insufficiency based on the father’s report of “not bearing primary responsibility for child rearing” combined with the mother’s report of “being the primary caregiver”. This method yielded a prevalence of fathering insufficiency of 30.4% [17].

Wang (2021) adopted a single-item measure to assess the prevalence of fathering insufficiency. This measurement approach is relatively simplistic. This study holds that a moderate level of paternal functioning should be taken as the reference criterion. The prevalence of fathering insufficiency can be defined as the proportion of individuals whose paternal functioning falls below this threshold. Furthermore, family structure—including dual-parent co-resident families, left-behind families and divorced families—exerts a significant impact on fathering insufficiency. Therefore, it is essential to further explore its prevalence across different family structures. Given that the sample of this study lacks population representativeness and has a limited sample size, the term proportion is uniformly adopted rather than prevalence in the empirical analysis.

### Association between fathering insufficiency and children’s mental health

Previous studies have explored the association between father absence or father-love absence and children’s mental health. This study adopts the concept of fathering insufficiency as a unified framework for review. First, fathering insufficiency evokes feelings of abandonment in children [18]. Second, children with fathering insufficiency suffer from more life adversities and mental strain. They often feel powerless to improve their living conditions, a state that gives rise to hopelessness [19], anxiety, and depression [14, 18, 20–22]. Third, fathering insufficiency is associated with a lack of adequate disciplinary education and supervision, which renders children more susceptible to delinquent behaviors, including truancy and theft [20]. Fourth, these children are vulnerable to social exclusion and peer marginalization, which intensifies their feelings of loneliness [23]. Fifth, children experiencing fathering insufficiency face higher risks of being bullied, which generates hostility and resentment toward others and society, and may even lead to aggressive and violent conduct [15, 24, 25]. Sixth, prolonged fathering insufficiency and cumulative life hardships can cultivate an individual’s sense of meaninglessness in life [26], which further induces non-suicidal self-injury [27] and suicidal ideation [28].

Existing research on the impact of fathering insufficiency on children’s mental health has two limitations. First, prior mental health assessments have predominantly focused on negative emotional and psychological outcomes, while largely neglecting positive emotions and psychological well-being. According to the dual-factor model of mental health, psychological well-being encompasses not only the absence of mental illness but also the presence of positive emotions and adaptive psychological functioning [29]. As the affective component of subjective well-being, emotional well-being (EWB) reflects an individual’s overall experience of positive and negative emotions [30], and serves as a crucial indicator of comprehensive mental health status [31]. Accordingly, the present study adopts emotional well-being as a key indicator to evaluate children’s mental health.

Second, prior research investigating the impacts of fathering insufficiency on children’s mental health has largely overlooked the contributions of maternal functioning. Zharima et al. (2026) demonstrated that maternal functioning exerts a certain compensatory effect on children experiencing fathering insufficiency [25]. However, their work relied exclusively on a qualitative interview design and lacked quantitative empirical evidence to validate its findings. Against this backdrop, this study integrates both paternal and maternal functioning into latent profile analysis, to further examine the relationship between fathering insufficiency and children’s emotional well-being.

### Purpose of this study

This study has three main objectives. First, it seeks to identify distinct types of fathering insufficiency using LPA, followed by an analysis of variance (ANOVA) to examine the differences among these profiles. Second, it aims to explore the proportion of fathering insufficiency across three family structures—dual-parent co-resident, left-behind, and divorced families. For each family structure, the proportion of each specific subtype of fathering insufficiency was computed by dividing the sample size of that subtype by the total sample size of the corresponding family structure. Third, it intends to compare differences in children’s emotional well-being across various latent profiles of fathering insufficiency.

## Method

### Procedure

The following steps were taken in the survey. First, we obtained ethical approval from the Academic Ethics Committee of the university affiliated to the first author. Second, six junior high schools were recruited as participants in the present study. This study adopted a quota sampling method that balanced two dimensions: geographic distribution (urban districts, county seats, and townships) and school quality (high-quality, medium-quality, and qualified). Equal quotas were set for each dimension (two schools per category). Subsequently, schools were contacted and those willing to participate were determined. These schools were distributed across Jiangsu, Fujian, and Henan provinces.

Third, we recruited teachers from participating schools who volunteered to assist with our research. We then provided them with training on the testing procedures via phone calls and WeChat instant messaging. Fourth, informed consent to participate was obtained from the parents or legal guardians of the participants—typically the grandparents of left-behind children—as the majority of participants were under the age of 16. Fifth, the formal survey was conducted in class. Participants were informed that their participation was voluntary, that they could withdraw at any time, that all responses would be kept confidential, and that they would receive a small gift upon completing the questionnaire. They were asked to provide assent after the procedures had been fully explained. After finishing the questionnaire, students were given a little gift.

### Participants

To screen out careless responses, two paired validity check items were embedded in the questionnaire: “I read each question carefully before giving my answer” and “I tick answers randomly without reading the questions carefully”. Questionnaires with obviously inconsistent responses to the two items were excluded. Ultimately, a total of 3266 valid questionnaires were retained. Students filled in a choice item about their parents’ status on the questionnaire: (a) both of my parents often accompany me; (b) one or both of my parents go out to work for more than 3 months; (c) one or both of my parents has/have passed away; or (d) my parents have divorced. Based on this item, 2710 students were from dual-parent co-resident families, 371 from left-behind families, 67 from parent(s)-bereaved families, and 118 from divorced families.

This study mainly explores quantitative differences in parental functioning. The death of a father or mother means individuals can no longer access the corresponding paternal or maternal functioning. Accordingly, 67 such participants were excluded, yielding a final sample of 3199 participants (1664 boys and 1535 girls). Their ages ranged from 11 to 17 years with a mean of 13.2 years (SD = 0.9). The sample consisted of 1583 adolescents from the first grade of junior middle school (Grade 7), 1348 from the second grade of junior middle school (Grade 8), and 268 from the third grade of junior middle school (Grade 9). Because students in the third grade of junior middle school (Grade 9) are busy preparing the entrance examination to senior middle school or technical secondary school in China, the school administrators often forbid them to take tests that are irrelevant to examination. Thus, fewer participants were recruited from the third grade of junior middle school compared with the first or second grade.

### Measures

Different paternal functions were measured using distinct subscales of the Inventory of Father Involvement (short version) [32], which was adapted into Chinese by Yin et al. (2012) [33]. Economic support was assessed using the 2-item Providing subscale (e.g., “My father accepts the responsibility for my financial support”). Daily care was measured with the 3-item Attentiveness subscale (e.g., “My father is involved in the daily or regular routine of taking care of my basic needs or activities such as feeding, driving me places and so on”). Emotional support was evaluated using the 3-item Time and Talking Together and 3-item Praise and Affection subscales (e.g., “My father praises me for something I have done well”). Academic support was assessed through the 3-item School Encouragement and 3-item Reading and Homework Support subscales (e.g., “My father helps me with my homework”). Discipline education was measured with the 3-item Discipline and Teaching Responsibility subscale (e.g., “My father sets rules and limits for my behaviours”). Future planning was evaluated using the 3-item Developing Talents and Future Concerns subscale (e.g., “My father plans for my future”). Participants were asked to report the frequency of their fathers’ parenting behaviours over the past year, on a scale ranging from 1 (*never*) to 5 (*always*). Confirmatory factor analysis yielded the following results: χ²/df = 2.86 ≤ 8.0, CFI = 0.949 ≥ 0.90, TLI = 0.940 ≥ 0.90, RMSEA = 0.044 ≤ 0.08 with a 95% confidence interval of [0.042, 0.047], and SRMR = 0.040 ≤ 0.08, indicating that the construct validity of the inventory is good in this study. The questionnaire demonstrated a Cronbach’s α coefficient of 0.919, and a McDonald’s ω coefficient of 0.921.

Consistent with previous studies [34–36], maternal functions were assessed with the modified version of the abovementioned questionnaire by replacing the word ‘‘father’’ with ‘‘mother’’. The model fit indices of confirmatory factor analysis were as below: χ²/df = 2.62 ≤ 5.0, CFI = 0.954 ≥ 0.90, TLI = 0.946 ≥ 0.90, RMSEA = 0.041 ≤ 0.08 with a 95% confidence interval of [0.040, 0.043], and SRMR = 0.037 ≤ 0.08, indicating that the construct validity of the revised questionnaire was good. The reliability of the questionnaire in this study was Cronbach’s α = 0.924, and McDonald’s ω = 0.925.

Emotional well-being (EWB) was assessed using 27-item Emotional Experience of Well-being Questionnaire [37], which measured fourteen positive emotions (e.g., sense of meaning, interest, hope, love, gratitude, satisfaction, relief) and thirteen negative emotions (e.g., anxiety, sadness, loneliness, emotional distress, depression, guilt, anger). Responses are made on a 5-point scale indicating the frequency that the participants experience the emotions in daily life ranging from 1 (*never*) to 5 (*always*) in the last year. The items in the dimension of negative emotion were reversed, and then added with the items of positive emotion to form the total score of emotional well-being. Higher scores indicate higher levels of emotional well-being. The results of confirmatory factor analysis (CFA) are presented as follows: χ²/df = 3.12 ≤ 8.0, CFI = 0.942 ≥ 0.90, TLI = 0.936 ≥ 0.90, RMSEA = 0.043 ≤ 0.08 with its 95% confidence interval ranging from [0.042, 0.045], and SRMR = 0.038 ≤ 0.08, indicating that the construct validity of the questionnaire was good. The Cronbach’s α coefficient of the questionnaire was 0.902, and the McDonald’s ω coefficient was 0.906.

### Statistical methods

Statistical software adopted in this study included SPSS 27.0 and Mplus 7.0.

#### Common method bias

Two ways are adopted to control common method bias: procedural control before data collection and statistical testing after data collection. Procedural control refers to a series of measures implemented during research design and questionnaire administration to mitigate common method variance at the source. In this study, respondents were clearly informed of the principles of voluntary participation, anonymity and confidentiality in the questionnaire instructions, and completion incentives were provided. These measures were taken to minimize response bias as much as possible.

The Harman’s single-factor test was employed to examine common method bias. All measurement items were entered into an unrotated exploratory factor analysis, and the severity of bias was assessed based on the variance explained by the first common factor. The diagnostic criterion is as follows: if the factor analysis extracts only a single common factor, or if the variance explained by the first common factor exceeds 40%, it indicates that the study suffers from severe common method bias [38].

#### LPA

Data preprocessing and assumption checks should be performed prior to LPA, including screening for missing values and outliers as well as normality tests of observed indicators. Missing values are examined via the menu path “Analyze → Descriptive Statistics → Frequencies”. The results showed that the 23-item Inventory of Father Involvement contained 5 missing responses, the 23-item Inventory of Mother Involvement had 3 missing responses, and the 27-item Emotional Experience of Well-being Questionnaire had 6 missing responses. These missing responses exhibited a random distribution pattern: (1) no participant had at least two missing responses; (2) all missing responses in the sample were scattered across different test items, with no item showing concentrated missing data. The formulas for calculating missing percentages are as follows: item missing percentage = number of missing responses for this item / sample size, and total missing percentage = total number of missing responses / (number of questionnaire items * sample size). A total of 3,199 participants were included in the present study. The item-level missing percentage stood at 0.03%. The total missing percentages were 0.007%, 0.004%, and 0.007%, respectively. Given the negligible missing percentage, this study employed the mode substitution method to handle missing values. This approach utilizes the measure of central tendency of the sample, thereby effectively preserving the full research sample without substantially altering the distributional properties of the original data.

The procedures for identifying outliers were as follows: (1) Select the menu path “Analyze → Descriptive Statistics → Descriptives”, and check the option “Save standardized values as variables (Z)”. A new variable prefixed with Z will be automatically generated in the SPSS Data View, which records the Z-score of each observation. (2) Data points with absolute Z-scores greater than 3.29 (i.e., exceeding 3.29 standard deviations from the mean) were classified as extreme outliers.

Normality tests can be conducted in three ways. (1) Skewness and kurtosis inspection. A skewness value close to zero indicates a more symmetric distribution, while a kurtosis value near zero suggests the distribution has a peak comparable to that of the normal distribution. A widely accepted empirical rule states that data can be deemed approximately normally distributed if the absolute value of skewness is less than 3 and the absolute value of kurtosis is less than 10. (2) Kolmogorov-Smirnov test. The data are considered consistent with a normal distribution when the Kolmogorov-Smirnov test yields non-significant results. Nevertheless, this test is highly sensitive to sample size. In large-sample scenarios, it tends to produce significant findings, leading to the rejection of the null hypothesis that the data follow a normal distribution. (3) Normal Q-Q plot, a pivotal graphical tool for normality diagnosis. Data are regarded as approximately normally distributed if all data points in the plot lie roughly along the diagonal reference line (the theoretical straight line).

Subsequently, tests for model suitability were carried out on the data, with a primary focus on the global identifiability of the model—namely, whether a unique solution exists for parameter estimates. Empirical studies have demonstrated that latent profile models estimated via maximum likelihood generally require a total sample size of at least 500, and the number of parameters to be estimated should not exceed one-tenth of the total sample size. The total sample size of the present study was up to 3,199, which was far above this threshold. Accordingly, the dataset contained sufficient statistical information to support model estimation.

All observed indicators included in LPA were calculated as the mean scores of all items within their corresponding dimensions. The model parameter settings were as follows. A mixture model was adopted for the parametric model, and robust maximum likelihood (MLR) was used as the parameter estimation method. A total of 1000 random starting values were set for the initial stage, and the top 200 solutions were retained for final-stage optimization.

The following criteria were used for deciding the best fitting model. (1) Akaike Information Criterion (AIC), Bayesian information criterion (BIC), and adjusted Bayesian information criterion (aBIC) [39]. Lower AIC, BIC, and aBIC estimates suggest better fitting models. (2) Lo–Mendell–Rubin likelihood ratio test (LMR) and bootstrapped likelihood ratio test (BLRT) [40]. The LMR and BLRT are used to test whether a k-class model fits the data significantly better than the (k − 1)-class model. A statistically significant result suggests that the k-class model is superior to the (k − 1)-class model; a non-significant result indicates no improvement in model fit for the k-class solution relative to the (k − 1)-class solution. (3) Entropy. Higher entropy values closer to 1 indicate greater classification accuracy. An entropy value greater than 0.80 implies that more than 90% of participants are correctly assigned to their corresponding latent profiles [41].

#### ANOVA

Before proceeding with ANOVA, the normality of the data was first examined. The detailed procedures for normality testing are described in Sect. 2.3.2. Levene’s test was adopted to test the homogeneity of variances. Post-hoc comparison methods were selected in accordance with the Levene test results: the Tukey-Kramer test was used when homogeneity of variances was assumed; otherwise, the Games-Howell test was applied in cases of unequal variances. Partial η² was used to assess the effect size of ANOVA. The effect size is considered small when partial *η*² < 0.059, medium when 0.059 ≤ partial *η*² < 0.138, and large when partial *η*² ≥ 0.138 [42, 43].

#### BCH correction method

This study examined inter-profile differences in emotional well-being—a distal continuous variable—among latent profiles characterized by fathering insufficiency. Hard classification based solely on individuals’ maximum posterior membership probabilities discards the classification uncertainty embedded in posterior probabilities. Unadjusted classification error would inflate mean differences in emotional well-being between profiles, narrow confidence intervals, and substantially raise the probability of false-positive test results, ultimately introducing bias into research inferences. Accordingly, the Bolck–Croon–Hagenaars (BCH) correction method was adopted in this study to conduct unbiased estimation and inter-group comparisons.

## Results

This study addressed three research objectives, with findings reported across five sub-sections. The second and third subsections provided evidence for Research Objective 1, the fourth subsection supported Research Objective 2, and the fifth subsection offers evidence for Research Objective 3.

### Test for common method bias

Harman’s single-factor test revealed that the unrotated exploratory factor analysis of all measurement items extracted 12 common factors. The variance explained by the first common factor was 24.996%, which was lower than the critical threshold of 40%, indicating no evidence of severe common method bias based on this diagnostic test.

### Latent profile analysis

Tests for outliers revealed no extreme outliers in the dataset. For normality testing, the maximum absolute skewness value was 0.610 (less than 3), and the maximum absolute kurtosis value was 0.452 (less than 10). All Kolmogorov-Smirnov (K-S) tests yielded significant results (*p* < 0.05), indicating that the data deviated from a strictly normal distribution in a rigorous statistical sense. Nevertheless, the Q-Q normal probability plots showed that all sample points were roughly distributed along the diagonal line, demonstrating that the data approximately followed a normal distribution.

The six paternal functions and the six maternal functions were incorporated into the latent profile analysis. Table 1 displays the fit indices of latent profile models and the sample sizes of each latent class under the most probable classification of each model. In terms of model comparison, the p-value of the Lo–Mendell–Rubin test (pLMR) for the 4-class model was 0.2722, indicating no statistically significant difference between the 4-class and 3-class models. However, the 3-class model yielded higher AIC, BIC and aBIC values and a lower entropy value relative to the 4-class model; thus, the 3-class model was discarded. The 5-class model was also rejected for the following reasons. First, the p-value of LMR for the 5-class model was 0.1420, indicating no statistically significant difference between the 4-class and 5-class solutions^1^. Second, the entropy value of the 4-class model was higher than that of the 5-class model, which reflected superior classification accuracy for the 4-class solution. Third, AIC, BIC and aBIC were descriptive fit indices calculated solely based on model log-likelihood and the number of parameters; they lacked the capacity to judge statistical significance. A lower index value merely signified better fit to the current sample data, yet it could not distinguish whether the improved fit captured genuine population heterogeneity or random sampling noise. Fourth, the 4-class model yielded larger sample sizes within each subgroup for subsequent analyses, thus offering greater statistical power. In a word, the 4-class model was selected as the optimal solution.

The best log-likelihood value of the 4-class model was − 43649.173, with 200 replications. The replication count exceeded 10, demonstrating stable model estimation results. The average posterior probabilities ranged from 0.912 to 0.949, far surpassing the conventional qualifying cutoff of 0.70. This indicated excellent separation between latent profiles and highly reliable classification.

**Table 1:** Latent class fit indices of paternal and maternal functioning

Model	AIC	BIC	aBIC	LMR(*p*)	BLRT(*p*)	entropy	Class size
1-class	103469.1	103614.7	103538.5	-	-	-	
2-class	92560.9	92785.6	92668.0	0.0000	0.0000	0.886	1500, 1699
3-class	89459.2	89762.8	89603.9	0.0000	0.0000	0.863	596, 1199, 1404
4-class	87424.3	87806.8	87606.6	**0.2722**	0.0000	**0.883**	189, 581, 1143, 1286
5-class	86139.0	86600.3	86358.9	0.1420	0.0000	0.861	180, 325, 665, 940, 1089

Note. Class sizes are based on most-likely class assignment

**Fig. 1:**
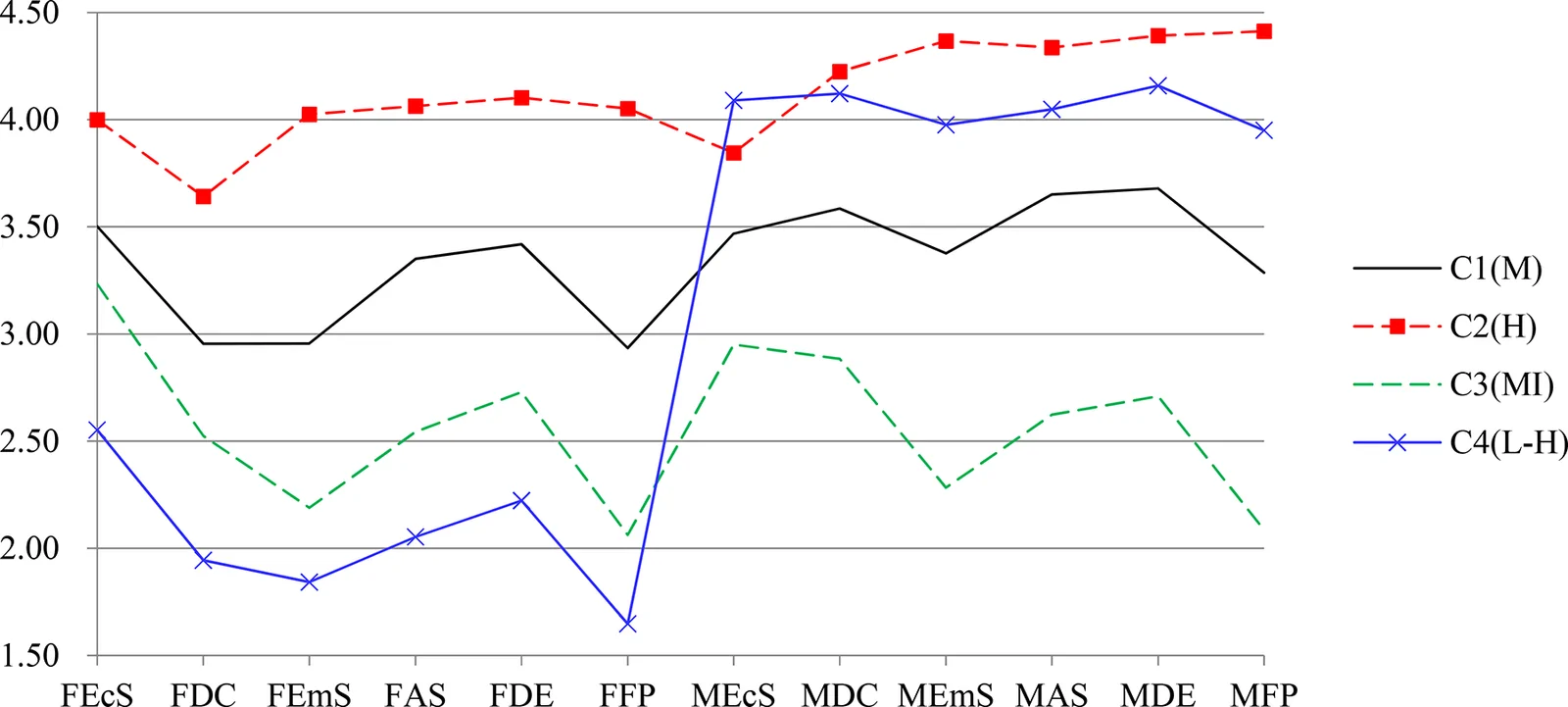
Latent profile of paternal and maternal functioning. Note. FEcS = father’s economic support, FDC = father’s daily care, FEmS = father’s emotional support, FAS = father’s academic support, FDE = father’s discipline education, FFP = father’s future planning, MEcS = mother’s economic support, MDC = mother’s daily care, MEmS = mother’s emotional support, MAS = mother’s academic support, MDE = mother’s discipline education, MFP = mother’s future planning

Conditional means, standard deviations (SDs), and latent class probabilities for the four-class model are presented in Table 2, while the latent profiles are illustrated in Fig. 1. The latent class proportion of Class 1 was the largest (0.402). The conditional means for the six paternal functions were about 3.0; and those for the six maternal functions were approximately 3.5. The conditional means in Class 1 were at a medium level among the four classes. Therefore, this class was labeled as moderate parental functioning class (M class).

The latent class proportion of Class 2 was the second largest (0.357). The conditional means for the six paternal functions were 4.0 or so; and those of the six maternal functions were around 4.0. The conditional means in Class 2 were at a high level among the four classes. Therefore, this class was labeled as high parental functioning class (H class).

In Class 3, the conditional means for the six paternal functions were approximately 2.5, and the means for the six maternal functions were also around 2.5. Compared with Class 1, the conditional means in Class 3 were at a lower level, and there exists a certain degree of parenting insufficiency. Therefore, this class was labeled as mild parenting insufficiency class (MI class).

This profile of Class 4 was characterized by the lowest conditional means across all six paternal functions with the lowest latent class proportion (0.059), indicating severe fathering insufficiency that we designated as fathering lack. The conditional means for the six maternal functions were as high as approximately 4.0. Therefore, this class was labeled as fathering-lack-high-mothering (L-H class).

Both the MI and L-H classes exhibited fathering insufficiency, with their latent class proportions being 18.2% and 5.9%, respectively. The sum of their latent class proportions was 24.1%.

**Table 2:** Conditional mean, SD and LCP of the 4-class model

Variables	C1		C2		C3		C4	
	M	SD	M	SD	M	SD	M	SD
FEcS	3.50	0.72	4.00	0.66	3.23	0.80	2.55	0.86
FDC	2.95	0.72	3.64	0.76	2.52	0.76	1.94	0.71
FEmS	2.96	0.66	4.03	0.65	2.19	0.72	1.84	0.70
FAS	3.35	0.66	4.06	0.58	2.54	0.79	2.05	0.79
FDE	3.42	0.70	4.10	0.64	2.73	0.85	2.22	0.91
FFP	2.93	0.78	4.05	0.71	2.06	0.76	1.65	0.66
MEcS	3.47	0.69	3.84	0.69	2.95	0.90	4.09	0.71
MDC	3.59	0.74	4.22	0.65	2.88	0.88	4.12	0.69
MEmS	3.38	0.68	4.37	0.57	2.28	0.72	3.98	0.75
MAS	3.65	0.64	4.34	0.54	2.62	0.85	4.05	0.66
MDE	3.68	0.68	4.39	0.58	2.71	0.84	4.16	0.67
MFP	3.29	0.73	4.41	0.62	2.09	0.73	3.95	0.85
N	1286		1143		581		189	
LCP	40.2%		35.7%		18.2%		5.9%	

Note. FEcS = father’s economic support, FDC = father’s daily care, FEmS = father’s emotional support, FAS = father’s academic support, FDE = father’s discipline education, FFP = father’s future planning, MEcS = mother’s economic support, MDC = mother’s daily care, MEmS = mother’s emotional support, MAS = mother’s academic support, MDE = mother’s discipline education, MFP = mother’s future planning, N = number of participants in each class, LCP = latent class proportions

### ANOVA results of fathering insufficiency

One-way ANOVA was further performed to examine the degree of fathering insufficiency across each dimension for the MI and L-H classes relative to the M class. As shown in Table 3, fathers in both the MI and L-H classes scored significantly lower than those in the M class across all paternal functions—namely, financial support, daily care, emotional support, academic support, discipline education, future planning, and total paternal functions. With the exception of financial support, which demonstrated a medium effect size, all other functions showed large effect sizes. It should be clarified that the paternal functioning profiles derived in this study were obtained by including maternal functioning indicators in the LPA model, instead of conducting separate clustering based solely on paternal functioning indices.

**Table 3:** ANOVA results of fathering insufficiency

Variables	M class(1)		MI class(3)		L-H class(4)		F	Post Hoc	Partial η²
	M	SD	M	SD	M	SD			
FEcS	3.50	0.72	3.23	0.80	2.55	0.86	137.58***	1 > 3>4	0.118
FDC	2.95	0.72	2.52	0.76	1.94	0.71	194.56***	1 > 3>4	0.159
FEmS	2.96	0.66	2.19	0.72	1.84	0.70	395.19***	1 > 3>4	0.278
FAS	3.35	0.66	2.54	0.79	2.05	0.79	441.12***	1 > 3>4	0.301
FDE	3.42	0.70	2.73	0.85	2.22	0.91	305.09***	1 > 3>4	0.229
FFP	2.93	0.78	2.06	0.76	1.65	0.66	414.18***	1 > 3>4	0.287
TPF	3.15	0.38	2.48	0.52	1.99	0.52	868.00***	1 > 3>4	0.458

Note. FEcS = father’s economic support, FDC = father’s daily care, FEmS = father’s emotional support, FAS = father’s academic support, FDE = father’s discipline education, FFP = father’s future planning, TPF = total paternal functions

### Proportion of fathering insufficiency across family structures

**Fig. 2:**
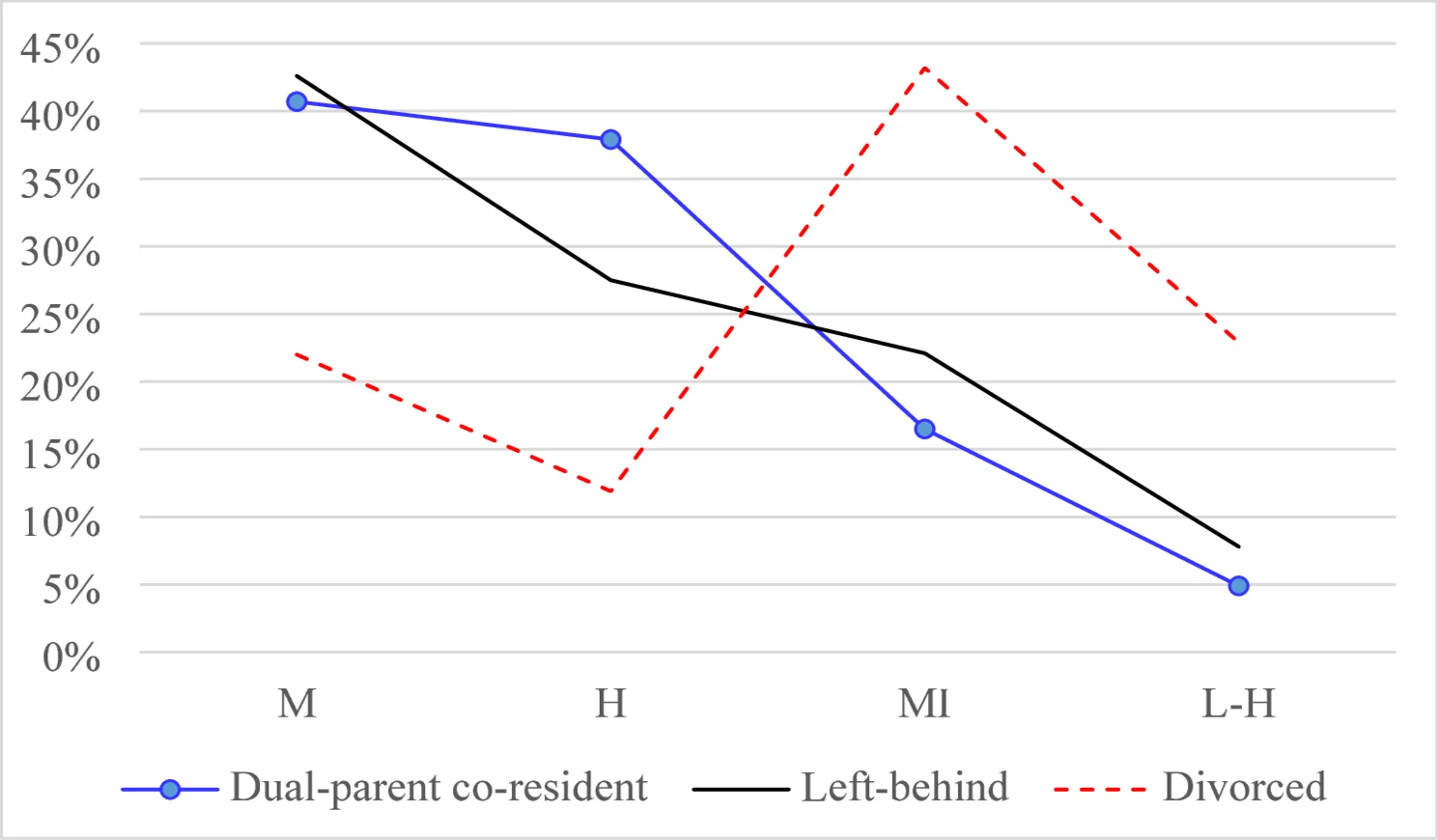
Proportion of fathering insufficiency across different family structures

The proportions of the MI and L-H classes within each family structure were calculated. As presented in Table 4; Fig. 2, the proportion of fathering insufficiency was the lowest (21.4%) in dual-parent co-resident families. This proportion was slightly higher in left-behind families (29.9%) than in dual-parent co-resident families. In contrast, divorced families exhibited a noticeably higher rate of fathering insufficiency (66.1%) compared to both dual-parent co-resident and left-behind households.

**Table 4:** Proportion of fathering insufficiency across different family structures

FS	M class		H class		MI class		L-H class		Total	
	*N*	*P*(%)	*N*	*P*(%)	*N*	*P*(%)	*N*	*P*(%)	*N*	*P*(%)
DPCRF	1102	40.7	1027	37.9	448	16.5	133	4.9	2710	100
LBF	158	42.6	102	27.5	82	22.1	29	7.8	371	100
DF	26	22	14	11.9	51	43.2	27	22.9	118	100

Note. The percentages are calculated within each family-structure row. FS = family structure, DPCRF = dual-parent co-resident families, LBF = left-behind families, DF = divorced families

### Relationship between fathering insufficiency and EWB

**Fig. 3:**
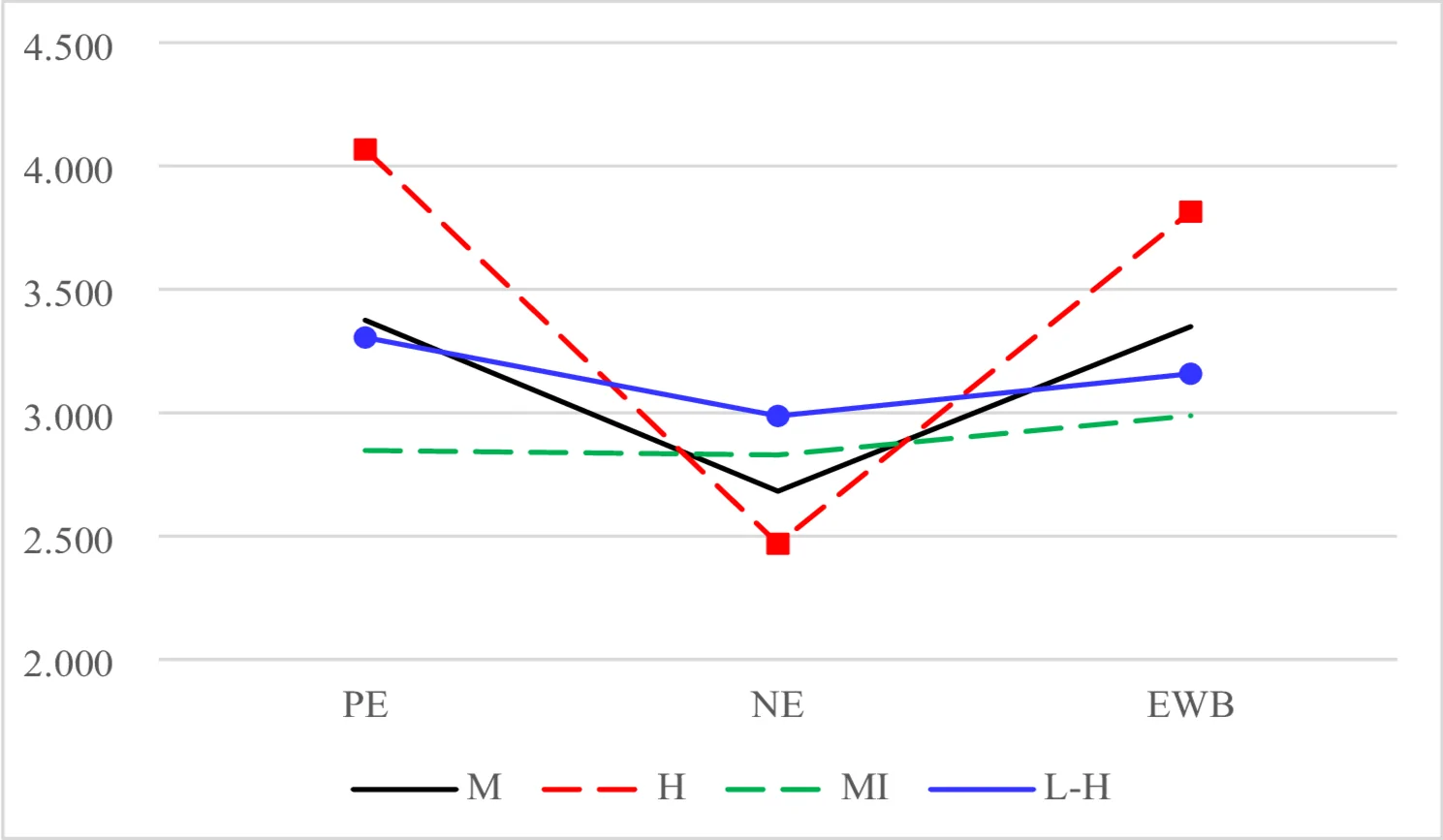
EWB across different types of fathering insufficiency

Following BCH correction, inter-profile differences in emotional well-being across latent profiles of fathering insufficiency are displayed in Table 5; Fig. 3. Children in the M class exhibited moderate positive emotions, low negative emotions, and relatively high emotional well-being. Those in the H class scored the highest on positive emotions, the lowest on negative emotions, and attained the highest level of emotional well-being. Children in the MI class had the lowest positive emotions and relatively high negative emotions, accompanied by the lowest emotional well-being. For children in the L-H class, their positive emotions remained at a moderate level while negative emotions peaked. Their emotional well-being was significantly lower than that of the M class yet significantly higher than that of the MI class.

**Table 5:** EWB across different types of fathering insufficiency

Variables	M(1)		H(2)		MI(3)		L-H(4)		χ²	Pair_com
	M	SE	M	SE	M	SE	M	SE		
PE	3.375	0.019	4.067	0.019	2.847	0.030	3.305	0.056	1365.7***	2> (1,4) > 3
NE	2.682	0.021	2.469	0.022	2.829	0.036	2.987	0.061	124.4***	2 < 1<3 < 4
EWB	3.349	0.016	3.815	0.017	2.988	0.026	3.158	0.047	837.1***	2 > 1>4 > 3

Note. PE = positive emotions, NE = negative emotions, EWB = emotional well-being, ***p < 0.001, Pair_com = pairwise comparisons, > indicates significantly greater (*p* < 0.05), < indicates significantly lower (*p* < 0.05), (x, y) indicates that there are no differences between x and y

## Discussion

### Relationship between fathering insufficiency and EWB

#### Types of fathering insufficiency

The first objective of this study was to identify types of fathering insufficiency through LPA. Based on twelve indicators (six paternal functions and six maternal functions), children were categorized into four classes: moderate parental functioning (M), high parental functioning (H), mild parenting insufficiency (MI), and fathering-lack-high-mothering (L-H). Fathers in the M class showed a medium level of paternal functioning, and accounted for the largest proportion (0.402) among all types. Accordingly, using the fathering level in the M class as the reference criterion of fathering insufficiency was justifiable at both the statistical and practical levels.

ANOVA results demonstrated that fathers in the MI and L-H classes displayed significantly lower levels of paternal functioning than their counterparts in the M class (Table 3), reflecting the presence of fathering insufficiency in these two groups. Based on four indicators—emotional support and leisure, discipline and punishment, school support, and daily care—Liu et al. (2026) conducted latent profile analysis and classified paternal functioning into three classes [44]: high parental functioning, moderate parental functioning, and low involvement. The low-involvement class aligns with the MI class identified in the present study. Nevertheless, the current study incorporated maternal functioning into latent profile analysis, which yielded an additional parenting profile labelled the L-H class.

The latent class proportion was 18.2% for the MI class and 5.9% for the L-H class, with a combined proportion of 24.1%. In other words, the overall proportion of paternal insufficiency among the sample was approximately 24%, which was lower than the 30.4% prevalence reported in a prior single-item measurement study [17]. Unlike the single-item assessment adopted in previous research, the present study evaluated parenting functioning across twelve dimensions and employed rigorous LPA and ANOVA approaches to identify fathering insufficiency. Therefore, the proportion estimate of fathering insufficiency derived from this study is considered more accurate and reliable.

#### Proportion of fathering insufficiency across different family structures

The second objective of this study was to examine the proportion of fathering insufficiency across different family structures. Table 4; Fig. 2 demonstrate that dual-parent co-resident families had the lowest proportion of fathering insufficiency; yet the rate still reached 21.4%. This finding offers empirical evidence that fathering insufficiency persists even when both parents are consistently present in the household. Moreover, it underscores the limitation of relying on such families as the reference standard for fathering insufficiency in conventional research, thereby indirectly supporting the rationale for adopting a moderate level of fathering as a new benchmark in this study.

Although the proportion of fathering insufficiency in left-behind families was higher than in dual-parent co-residing families, the difference was not statistically significant. This phenomenon may be explained from two perspectives. First, a father’s migration for work helps improve the household’s financial situation, which not only reflects his fulfillment of a core family function but also establishes the material foundation for performing other paternal roles. Second, China’s highly developed information and communication technologies, such as video calls and instant messaging, have effectively enhanced the quality of parent-child interactions across geographical distances, thereby partially compensating for the lack of emotional support caused by physical separation [45].

However, the proportion of fathering insufficiency in divorced families was notably high. Specifically, the proportion of the MI class reached 43.2% (Table 4). This may be attributed to several factors following divorce. First, approximately three-quarters of fathers lose custody of their children [4, 5], resulting in diminished contact and reduced paternal functioning. Second, mothers, who are primarily responsible for caring for the child alone, face substantial economic and daily caregiving burdens [5, 46], which makes it difficult for them to adequately invest in other areas such as emotional support, academic guidance, and future planning. Moreover, the number of remarried individuals in China has been steadily increasing [47]. During the process of forming a stepfamily and thereafter, parental investment in children tends to decrease as well [48]. Therefore, a large proportion of divorced families fall into the MI class.

Furthermore, divorced families exhibit a relatively high proportion of L-H class (Table 4). This pattern may be explained by the fact that some non-custodial fathers, who no longer live with their children after divorce, tend to exhibit very low levels of paternal functioning. In contrast, some mothers are relieved from marital conflicts and distress, embark on a positive new life with enhanced life satisfaction [49], and thereby become better equipped to compensate for children’s father absence.

#### EWB across different types of fathering insufficiency

The third objective of this study was to compare differences in emotional well-being among children with different types of fathering insufficiency. As illustrated in Table 5; Fig. 3, the relationship between combined paternal and maternal functioning profiles and children’s emotional well-being exhibits the following characteristics. The first characteristic was consistency. For the three classes of M, H, and MI, higher parental functioning across economic support, daily care, emotional support, academic support, disciplinary education, and future planning was associated with greater satisfaction of children’s multifaceted needs, including physical, emotional, relational, and esteem needs. Correspondingly, children reported higher levels of positive emotions, lower negative emotions, and superior emotional well-being. In contrast, low levels of both paternal and maternal functioning were adversely related to children’s need fulfillment, which in turn led to poorer emotional well-being.

The second characteristic was compensation. For children in the L-H class, paternal functioning was very low, yet maternal functioning remained high. Their diverse psychological needs could be satisfied to a certain extent, resulting in significantly higher positive emotions compared with adolescents in the MI class. In other words, maternal functioning exerted a certain compensatory role, which aligned with the findings of prior research [25]. Nevertheless, extensive maternal functioning could not fully substitute paternal functioning. Due to severe lack of paternal functioning, some needs of the child were difficult to fulfill, which not only triggered intense feelings of abandonment [18], anxiety and depression [14, 18, 20–22], but also brought about loneliness [23], hostility and resentment [15, 24], resulting in the highest level of negative emotions. Overall, the emotional well-being of children in the L-H class was significantly lower than that of children in the M class, yet significantly higher than children in the MI class. This indicated that high levels of maternal functioning could sustain and boost children’s positive emotions, yet failed to effectively alleviate negative emotions stemming from severe fathering lack. This finding provided evidence that compensatory parenting contributes positively to children’s emotional well-being.

### Highlights

This study yielded the following highlights. First, it proposed the term “fathering insufficiency” to describe insufficiency in paternal functioning, moving beyond a mere focus on physical absence of fathers.

Second, this study adopted a moderate level of paternal functioning as the criterion for defining fathering insufficiency, which aligned more closely with statistical principles compared to the conventional approach of using dual-parent co-residence as the criterion.

Third, this study identified two subtypes of fathering insufficiency via latent profile analysis: mild parenting insufficiency and fathering-lack-high-mothering classes, which further refined the heterogeneous manifestations of insufficient paternal functioning.

Fourth, the study examined the proportion of fathering insufficiency across different family structures and presented three key findings: fathering insufficiency was empirically confirmed in dual-parent co-resident families, challenging the assumption that co-residence ensured parental functioning; its proportion in left-behind families was only marginally elevated, cautioning against overestimation; and it was most severe in divorced families, calling for prioritized attention.

Finally, this study integrated maternal functioning into the analytical framework examining how fathering insufficiency was negatively related to children’s emotional well-being. There were two core patterns underlying the relationship between profiles of combined paternal and maternal functioning and children’s emotional well-being: consistency and compensation. For adolescents in the M, H, and MI classes, greater parental functioning was linked to higher emotional well-being. In contrast, among adolescents in the L-H class, high maternal functioning was positively associated with their positive emotions yet only weakly related to the alleviation of negative emotions. Although their emotional well-being remained lower than that of the M class, it was significantly higher than the MI class. This finding suggested that compensatory maternal functioning exhibited a modest positive relationship with children’s emotional well-being.

### Practical implications

It is the universal desire of most parents to help their children be happy and joyful. How can we enhance children’s emotional well-being? Increasing paternal functioning and reducing fathering insufficiency are important pathways to achieving this goal. Specifically, the following approaches can be adopted.

First, it is essential to strengthen family education, help both parents recognize the importance of parental functioning and the potential negative impacts of fathering insufficiency, thus enhancing fathers’ sense of responsibility and the cooperative atmosphere within the family. Nowadays, many schools in China have launched “parenting classes”, which can be utilized to carry out family education on fathering insufficiency.

Second, fathers should make more flexible use of fragmented time in daily life and leverage modern information technology (such as video calls, messaging interactions) to strengthen emotional bonds with their children and increase their academic support.

Third, parents should make every effort to handle marital conflicts well, improve marital satisfaction, and think twice before opting for divorce with children’s well-being in mind. Conflicts are bound to arise over the course of a long marriage. When they do, it is better to resort to negotiation rather than coercion or avoidance. Because marital conflicts tend to be complex and sensitive, couples may find it helpful to attend counseling sessions and work with a psychological counselor to address their issues. Unless the marriage involves serious conflict such as domestic violence, coercive control, abuse, divorce should not be readily adopted as a solution to marital discord.

Finally, in cases where parents are divorced, fathers should take the initiative to maintain regular communication and companionship with their children. And mothers should prioritize children’s physical and psychological development, proactively support father-child visits and interactions, and work with fathers to build sound parent-child relationships.

### Limitations and future directions

Despite its contributions, some limitations of this study should be acknowledged. First, quota sampling, a non-probability sampling technique, was used to select schools in the present study. Future research may adopt probability sampling for school recruitment to greatly improve sample representativeness and strengthen the generalizability of research findings.

Second, although 3,199 children were surveyed in this study, the sample sizes of left-behind children (*n* = 371) and single-parent children (*n* = 118) were relatively small. Future research could adopt larger sample sizes to more accurately estimate the proportion of fathering insufficiency across diverse family structures and their intergroup differences.

Third, this study only included junior high school students. Children of different age groups have varying needs for parental care; therefore, the impact of fathering insufficiency may differ across developmental stages. Future studies could investigate fathering insufficiency and its effect on mental health among elementary, senior middle school, and college students.

Fourth, as children grow older, their need for autonomy increases. Thus, providing autonomy support is also an important paternal function. Building on this study, future research could further explore how insufficient paternal autonomy support affects children’s mental health.

Fifth, this study only examined fathering insufficiency among Chinese children. Existing cross-ethnic research has shown that White fathers demonstrate significantly higher levels of paternal functioning than Black fathers [50]. Furthermore, in countries with a stronger patriarchal cultural climate, both mothers and fathers generally undervalue the importance of paternal participation in child-rearing, resulting in correspondingly lower paternal functioning [51]. Future research could expand the sample scope to compare the varying degrees of fathering insufficiency across diverse cultural and regional contexts, and further explore its differential mechanisms influencing children’s emotional well-being.

Sixth, it should be noted that the profiles derived from LPA are not deterministic groups but probabilistic structures constructed based on each individual’s probability of belonging to a specific profile. In other words, there is a certain degree of uncertainty regarding class membership. Future research could recruit more samples to further verify the stability of the latent profile structure identified in the present study.

Seventh, this study solely adopted self-report methodology, which carries inherent response biases including social desirability, self-concealment, and response sets. Reliance on a single data source also tends to trigger common method bias and results in insufficient ecological validity. Future research may employ multi-source assessment by incorporating other-reports from parents and teachers or direct behavioral observations. Cross-verification of diverse data can mitigate common method bias and enhance the ecological validity of measurements.

Eighth, this study adopted the moderate paternal functioning as the reference criterion for fathering insufficiency based on the statistical distribution characteristics of the sample data. It should be noted that this criterion is merely an empirically derived classification specific to the present sample and does not possess general applicability as a clinically validated or normative threshold. Future research is encouraged to introduce external criteria for cross-validation to further examine the predictive validity of this criterion, or to develop more refined assessment standards.

Ninth, this study only adopted cross-sectional survey data, which makes it difficult to clearly reveal the longitudinal temporal association between fathering insufficiency and children’s emotional well-being. Follow-up research may adopt a longitudinal tracking design to further clarify and verify the relationship between the two variables.

## Conclusion

Father absence—defined as physical separation between fathers and their children—does not necessarily entail severe deficits in paternal functioning. This study proposed the term “fathering insufficiency” to accurately describe inadequate paternal functioning, and adopted moderate paternal functioning as the reference criterion for evaluating fathering insufficiency.

Latent profile analysis classified adolescents into four groups: moderate parental functioning (M), high parental functioning (H), mild parenting insufficiency (MI), and fathering-lack-high-mothering (L-H), with the MI and L-H groups representing different patterns of fathering insufficiency.

Among adolescents in the L-H class, high maternal functioning may partially offset the effects of father absence, showing a positive association with children’s emotional well-being. Though adolescents in the L-H class reported lower emotional well-being than their M-class counterparts, their emotional well-being was notably higher than those in the MI class.

To boost children’s mental health, parent‑training programs shall deliver targeted education to reduce fathering insufficiency. Fathers should utilize fragmented time and digital tools to strengthen paternal functioning, while parents need constructive marital conflict resolution and prudent divorce decision‑making.

## Supplementary Information

Below is the link to the electronic supplementary material.

Supplementary Material 1

## Data Availability

Data are provided in the supplementary file.

## Footnotes

1 We also plotted the latent profiles for the 5-class model and compared them with those of the 4-class model. Both models retained the mild parenting insufficiency (MI) and fathering-lack-high-mothering (L-H) classes, which were the primary foci of this study. The main difference between the two models was that the high parental functioning (H) class in the 4-class model was further divided into two subcategories in the 5-class model. However, this subdivision was not the focus of our research. Therefore, we still adopted the 4-class model.
